# Dose-Related Analysis in Percutaneous Central Venous Catheters Insertion: Experience of a Pediatric Interventional Radiology Center

**DOI:** 10.3390/children9050679

**Published:** 2022-05-06

**Authors:** Gian Luigi Natali, Giulia Cassanelli, Claudia Polito, Vittorio Cannatà, Marco Martinelli, Massimo Rollo

**Affiliations:** 1 Imaging Department, Bambino Gesù Children’s Hospital, IRCCS, 00165 Rome, Italy; gianluigi.natali@opbg.net (G.L.N.); marco.martinelli@opbg.net (M.M.); massimo.rollo@opbg.net (M.R.); 2 Medical Physics Department, Bambino Gesù Children’s Hospital, IRCCS, 00165 Rome, Italy; claudia.polito@opbg.net (C.P.); vittorio.cannata@opbg.net (V.C.)

**Keywords:** central venous catheter, children, safety, ionizing radiation, ultrasound, dose area product, pediatric patients, newborns, preterm neonates, effective dose

## Abstract

Background: There are many techniques for long-term central venous catheter (CVC) placement, but none of them are specific for pediatric patients or focused on the delivered dose of ionizing radiation. Materials and Methods: This retrospective study examined a sample of pediatric patients who received percutaneous long-term CVC positioning in a tertiary care pediatric hospital. Effective dose, dose-area product (DAP) and length of time of exposition during the procedure were determined, using an appropriate technical procedure, exam and program set of the angiograph, and compared with an unpaired t-test analysis. Results: The study included 1410 enrolled patients, with a median age of 10 years (range 0.2–18 years), between 2016 and 2019. In 2016 (318 pts), the mean effective dose was 0.13 mSv and the mean DAP dose was 18.95 µGy/m^2^ In 2017 (353 pts), the mean effective dose was 0.11 mSv and the mean DAP dose was 17.26 µGy/m^2^. In 2018 (351 pts), the mean effective dose was 0.05 mSv and the mean DAP dose was 7.23 µGy/m^2^. In 2019 (388 pts), the mean effective dose was 0.02 mSv and the mean DAP dose was 3.10 µGy/m^2^. Conclusions: Medical and technical expertise led to a remarkable reduction in the radiation dose. Therefore, the authors’ hypothesis is that US- and fluoroscopy-guided percutaneous long-term CVC insertion technique is safer, more cost-effective and lower in terms of radiation exposure if correctly applied, compared to surgical or percutaneous by direct puncture techniques.

## 1. Introduction

During the last decade, the use of central venous catheters (CVC) in the treatment of multiple pathologies, mostly in patients with oncologic diseases, has become almost mandatory.

The main focus has always been on improving the percutaneous long-term CVC positioning technique to reduce patient radiation exposure as much as possible, and to shorten the learning curve for young interventional radiologists.

It has been stated that pediatric patients have greater radiosensitivity than adults. Moreover, an increased radiation exposure time and often a previous history of malignancy can further increase the risk of late radiation effects.

Many published studies on the radiation dose in CVC positioning have reported non-uniform results between adult and pediatric populations.

The objective of this study, which focused on dose-related analysis in 1410 procedures, is to avoid confusion and mistakes when choosing among the different approaches in CVC insertion.

Based on an improved learning curve, the less irradiating and most suitable technique for pediatric patients is the US- and fluoroscopic-guided percutaneous technique.

## 2. Materials and Methods

Bambino Gesù Children’s Hospital Ethics Committee granted that written informed consent was not required because it was unnecessary according to the Declaration of Helsinki.

A retrospective study of a pediatric sample was carried out at Bambino Gesù Children’s Hospital to evaluate the radiation doses to patients undergoing long-term percutaneous CVC positioning procedures performed by three pediatric interventional radiologists, between 2016 and 2019, on a total of 1410 pediatric patients.

The devices inserted during this period were open-ended, single or double-lumen tunneled catheters. We excluded from our study port-a-caths and peripherally inserted central catheter (PICC) positioning.

For each procedure, we determined the age, weight of the patient, DAP dose, fluoroscopy time and effective dose.

**Figure 1:**
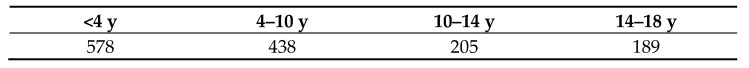
Age distribution of patients.

The median age of the enrolled patients was 10 years (range 0.2–18 years), as observed in Figure 1.

More than 66% of the insertion procedures were carried out on young patients, under 10 years of age.

The median weight of the included patients was 34 kg (range 2–67 kg).

In order to evaluate the optimization of radiation protection for patients undergoing procedures involving ionizing radiation, they were stratified into five age groups following the indications of the European Guidelines on Diagnostic Reference Levels (DRLs) for Paediatric Imaging [1]. A statistical analysis was carried out using Microsoft Excel (version 16.0.13001.20266).

Data were compared with an *unpaired t-test analysis*.

A *p* value below 0.05 was considered statistically significant.

All the insertion procedures were carried out under the guidance of a biplane angiography system (Artis Zee, Siemens, Erlangen, Germany) equipped with a large flat-panel (30 × 40 cm^2^).

The X-ray imaging system had a declared inherent filtration of ≥2.5 mmAl and a tube voltage up to 125 kV.

All the procedures were performed in AP projection with the same low-radiation exposure protocol, in order to guarantee a standard for all the X-ray examinations (70 kV, variable mA, additional filtration 0.9 mmCu).

As recommended by the European guidelines, all the latest radiological devices have to provide information about the patient’s dose.

In digital X-ray equipment, this indication is often given as DAP and is stored in the DICOM header files and/or in a dose-structured report file.

In order to provide the DAP indication, the angiograph is equipped with a calibrated ionization chamber DAP meter, located in the tube housing.

The system provides a readout of the cumulative DAP for every radiological procedure.

Patient and exposure data were used as input for the Monte Carlo simulation, using PCXMC software [2].

This simulation program allows one to calculate effective € and equivalent (*H_T_*) organ doses, using as input information the patient data (age, weight and height) and examination parameters, such as tube voltage, inherent and additional filtration, projection, focus to skin distance (FSD), field size, skin entry point of the central beam axis and input dose quantity (e.g., DAP) [Appendix A].

In this way, it is possible to simulate the real irradiation condition for each patient.

*E* and *H* are protection quantities, defined by the International Commission on Radiological Protection (ICRP) as quantities used “to ensure that the occurrence of stochastic health effects is kept below unacceptable levels and that tissue reactions are avoided” [3].

Preoperative US examination to identify the most suitable vein was performed for all the patients.

Usually, the right internal jugular vein is chosen (IJV access is the best option, due to its less tortuous anatomy; if parietal thrombi are present or anatomic problems are identified at the confluence in the anonym vein, the left-side approach is advised).

The angiograph has to be pre-set by the radiographer, using small FOV without any magnification and with 0.5/s frame rate fluoroscopy.

The procedure starts performing by creating a right (or left) sided latero-thoracic subcutaneous tunnel, followed by US-guided IJV puncture and venous cannulation with a wire and then, and only at this point, the first fluoro-shot.

A second fluoro-shot follows the peel away introduction and helps to measure the catheter length.

The last fluoro-shot is then obtained to check and store the final CVC position.

US and fluoroscopic guidance are mandatory to reduce the number of attempts at vein cannulation, to avoid inadvertent arterial puncture or scarring of the surrounding tissues, and to check the correct catheter position, which are all very relevant points in a mini-invasive approach, especially in pediatric patients.

## 3. Results

The retrospective study, carried out between 2016 and 2019, enrolled 1410 pediatric patients (Table 1), with a median age of 10 years (range 0.2–18 years).

In 2016 (318 patients), the mean effective dose was 0.13 mSv and the mean DAP dose was 18.95 µGy m^2^.

In 2017 (353 patients), the mean effective dose was 0.11 mSv and the mean DAP dose was 17.26 µGy m^2^. In 2018 (351 patients), the mean effective dose was 0.05 mSv and the mean DAP dose 7.23 µGym^2^.

**Figure 2:**
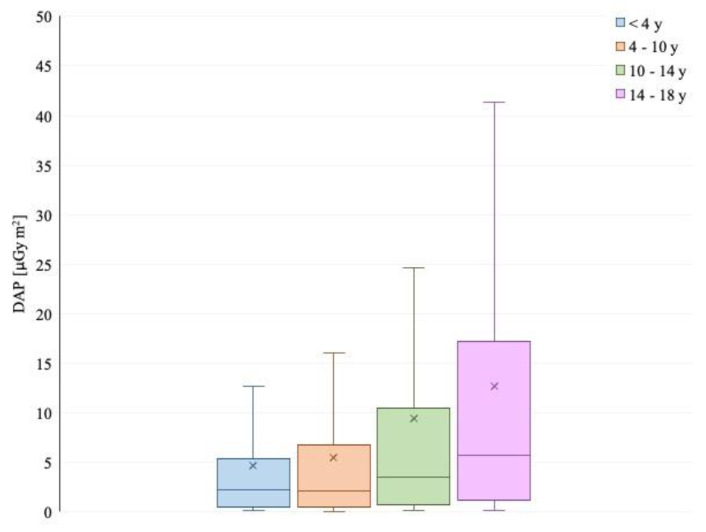
Total dose area product (µGy/m^2^) in different patient age groups.

**Figure 3:**
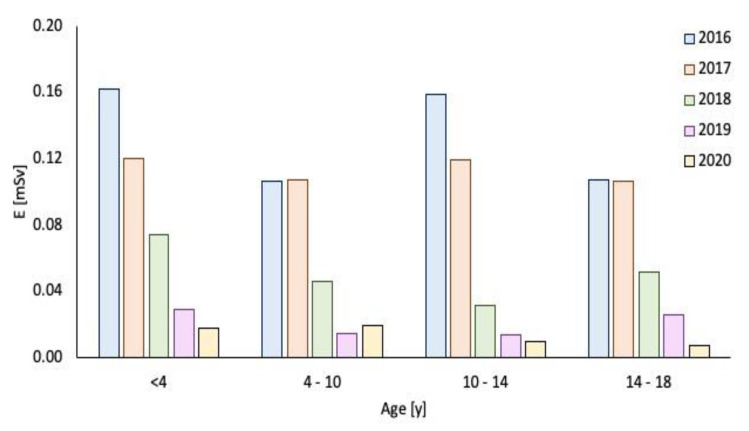
Total radiation dose (mSv) in different patient age groups.

**Figure 4:**
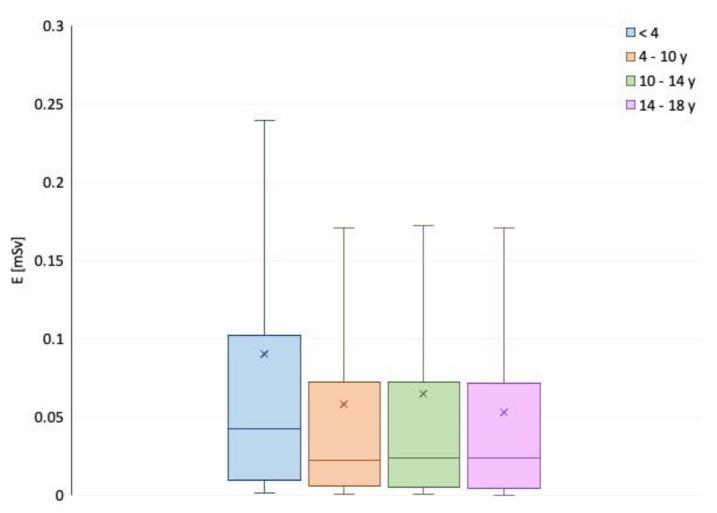
Radiation dose trend from 2016 to 2019 in different patient age groups.

**Figure 5:**
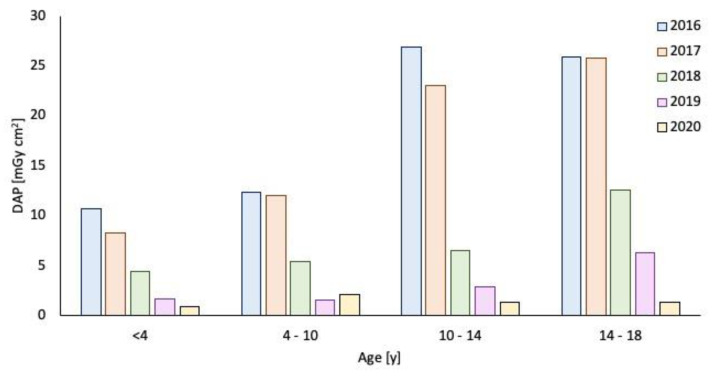
Dose area product trend from 2016 to 2019 in different patient age groups.

In 2019 (388 patients), the mean effective dose was 0.02 mSv and the mean DAP dose was 3.10 µGy m^2^. The DAP and effective dose values were significantly reduced in 2018–2019 compared to 2016–2017 (*p* < 0.05), as observed in Figure 2, Figure 3, Figure 4 and Figure 5.

**Figure 6:**
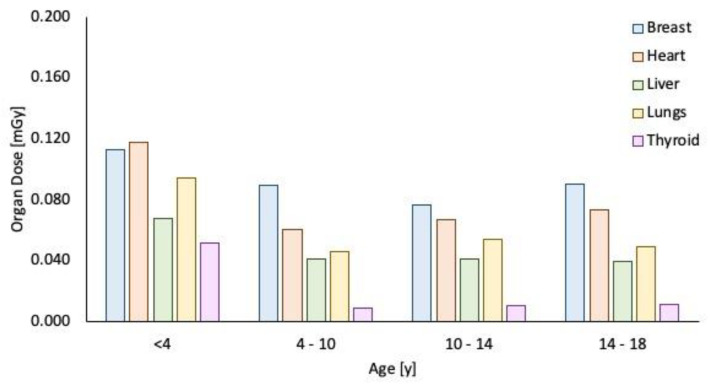
Organ dose (mGy) in different patient age groups.

In addition, the mean doses to organs fully or partially contained in the field of view (e.g., heart and thyroid, respectively) were significantly reduced (Figure 6).

The median fluoroscopy time per procedure in 2016 and 2019 was 0.28 s and 0.09 s, respectively, showing a statistically significant decrease of 67% (*p* < 0.05).

## 4. Discussion

Many techniques for long-term central venous catheter (CVC) placement are widely used in adults, but none of them are specific for pediatric patients or focused on the delivered dose of ionizing radiation.

Storm et al. analyzed data including fluoroscopy time, cumulative effective dose and dose-area product (DAP) in an adult population and relating to around 1000 instances of 6 different venous access procedures over 6 years, and found that no procedure yielded a cumulative dose of more than 950 mGy, or a peak skin dose of more than 760 mGy [4].

Chida et al. examined the relationship between fluoroscopy time and DAP in 200 consecutive adult interventional procedures, measuring patient skin dose, fluoroscopy time and DAP [3].

They observed that DAP is the gold standard for calculating radiation dose and that weight multiplied by fluoroscopic time should only be used if DAP cannot be applied.

Friend et al. analyzed a sample of 505 pediatric patients who received surgically implanted venous access devices (totally implanted and tunneled), collecting data on DAP and fluoroscopy time [5].

A small portion of this sample received a percutaneous approach, resulting in a longer DAP; however, despite this and the authors’ assumption that open surgical cut-down insertion significantly reduced radiation exposure compared to per-cutaneous techniques, clinical adverse effects or complications are considered minimal and the resultant radiation risk is estimated to be very low [5].

As listed below, various approaches to CVC positioning have been tested during the last few decades to try to determine the easiest and safest technique. In addition, particularly in pediatric patients, the main focus is to use a minimally invasive approach with minimal radiation exposure, since a high percentage of the smaller patients who need long-term CVC are affected by oncologic diseases and have a higher radiosensitivity.

The open surgical cut-down (OSC) technique has always been the most popular approach in adult populations and has also been widely used in the past in pediatric populations.

Avanzini et al. described the transition from the traditional open surgical cut-down procedure to US-guided percutaneous CVC insertion in 188 pediatric patients, focusing on the learning curve and related complications [6,7,8,9]. They stated that this shift presents a challenging learning curve that is generally associated with high rates of complications, which decrease progressively once a good expertise in US guidance and the percutaneous technique has been acquired.

The percutaneous approach by direct venipuncture of a major central vein was considered the safest technique by Pittiruti et al. [2] Their study reported on the insertion of medium and long-term CVC, Port-a-caths, as well as tunneled and non tunneled catheters, analyzing a mixed adult and pediatric population. They affirmed that a “low lateral” approach to the IJV may be the easiest and safest technique for percutaneous insertion of CVC, and that the only 100% safe method for avoiding PNX is to avoid direct puncture of the subclavian vein.

Many reviews, mainly by anesthesiologists that take care of central line positioning in PICUs, describe endocavitary-ECG control of the catheter position based on the principle that the catheter itself, once filled with normosaline and connected to a normal electrocardiograph, acts as an exploring electrode, which can monitor the progress of the catheter introduced percutaneously or surgically [2,10].

The main disadvantage of this technique is that radiological monitoring is considered mandatory to avoid CVC placement errors.

Other authors, such as Schummer et al., assert that the start of an increase in P wave size does not correspond with the entrance of the right atrium, showing the inability to distinguish between venous and arterial catheter position [11].

Moreover, when there are vascular anomalies, previous or recent thrombosis or post-surgical scars, the possibility to pass the tip of the wire through the atrium is never guaranteed using this technique.

In comparison to other studies in pediatric patients, this retrospective analysis, conducted on a wide and unique pediatric sample of 1410 patients, led to the conclusion that long-term central venous catheter (CVC) positioning, performed by a dedicated pediatric interventional radiology team, is highly recommended. Ultrasound and fluoroscopic guidance shows a relevant reduction in terms of the effective dose obtained by adopting an accurate technique and preset (0.5/s frame rate, no magnification, no anti scatter grids) [2,3,4,5,6,11,12].

The obtained results show that the effective doses to the young patients are extremely low, even to radiosensitive organs, such as the thyroid, which are often included in the field of view, especially in neonates or small children [13].

These values are comparable to those of a child’s chest X-ray (up to 0.1 mSv), which is equivalent to about 10 days of exposure to the natural background radiation to which we are all exposed during our lifetime [1,12,13,14,15,16,17,18,19].

## Figures and Tables

**Table 1:** Number of procedures per year.

	*<4 y*	*4–10 y*	*10–14 y*	*14–18 y*
*2016*	147	91	48	32
*2017*	142	112	52	47
*2018*	141	107	50	53
*2019*	148	128	55	57

## Data Availability

Data sharing not applicable, No new data were created or analyzed in this study. Data sharing is not applicable to this article.

## Appendix A

In the present study, the following quantities have been evaluated:

- Dose-area product (*DAP*) is the quantity usually used in radiographic and fluoroscopic procedures to provide indications about the radiation dose delivered to a patient. It is defined as the product of the surface area of the patient that is exposed to radiation at the skin entrance and radiation dose at this surface. It can be calculated using the following equation:
  $$DAP=D_{air}\times A$$
  where *D_air_* is the absorbed dose to air at the *DAP* meter position and *A* is the irradiated area.
- Equivalent dose (*H_T_*) measures the biological damage caused by the absorption of radiation by an organ or tissue. It is defined as the mean absorbed dose deposited in body tissue or organ *T* multiplied by the radiation weighting factor (*w_R_*) and can be calculated using the following equation:
  $$H_{T}=\sum_{R}w_{R}D_{T,R}$$
- The effective dose, *E*, is defined by the weighted sum of tissue equivalent doses and can be calculated using the following equation:
  (A1) $$E=\underset{T}{\sum }w_{T}H_{T}=\sum_{T}w_{T}\sum_{R}w_{R}D_{T,R}$$
  where *w_T_* is the tissue weighting factor for tissue *T*.
